# FEC Check: Development of a decision support tool to aid interpretation of gastrointestinal nematode faecal egg counts in sheep

**DOI:** 10.1002/vetr.70221

**Published:** 2026-01-06

**Authors:** Eilidh Geddes, Andrew Duncan, Kate Lamont, Jade M. Duncan, Dave J. Bartley, Neil Sargison, Fiona Kenyon, Lynsey A. Melville

**Affiliations:** ^1^ Moredun Research Institute Penicuik UK; ^2^ University of the Highlands and Islands Inverness UK; ^3^ Centre for Epidemiology and Planetary Health Department of Veterinary and Animal Science Scotland's Rural College Inverness UK; ^4^ Royal (Dick) School of Veterinary Studies University of Edinburgh Edinburgh UK

**Keywords:** decision support tool, faecal egg count, gastrointestinal nematodes, sheep

## Abstract

**Background:**

Gastrointestinal nematode infections are ubiquitous in grazing livestock worldwide impacting animal health and production. Faecal egg count (FEC) is an accessible diagnostic test that can guide the need for treatment. However, interpretation of FECs can be challenging.

**Methods:**

A prototype decision support tool (DST) was developed using a ‘traffic light’‐style gradient of potential clinical impact on sheep FEC results. Focus groups were conducted with farmers, livestock advisors and veterinary clinicians to examine the barriers to FEC uptake and provide feedback on the prototype tool.

**Results:**

Barriers to uptake for FEC testing included timeliness of reporting, lack of perceived need and knowledge gaps. The DST was well received at all focus groups, with simplicity and ease of use identified as key principles to drive uptake. At the 12 months post‐launch, the DST had 1916 users.

**Limitations:**

Engagement with stakeholders with less familiarity with FECs may improve usability for a wider audience.

**Conclusion:**

The final DST developed here represents a practical resource to improve the interpretation of FEC results reported by farmers and other stakeholders. The initial uptake observed within the first year since launch is promising for the wider adoption of evidence‐based parasite management.

## INTRODUCTION

Gastrointestinal nematode (GIN) infections are ubiquitous in grazing livestock worldwide, impacting on animal health, production efficiency and greenhouse gas emissions.^1^ GINs are generally controlled using broad‐spectrum anthelmintics, for which five classes of active compounds are licensed for use in sheep in the UK.^2^ Over‐reliance on anthelmintics for many years has led to the development of resistance in several GIN species and threatens the future sustainability of sheep production.^3^, ^4^, ^5^ To safeguard chemical GIN control for future use farmers, livestock advisors and veterinarians must find a way to reduce the selection pressure on anthelmintics while maintaining productivity in grazing stock. To achieve this balance, farmers are urged to only treat animals when they will benefit from the intervention in addition to implementing other herd or flock management strategies (e.g., evasive or co‐grazing). However, a key challenge for producers is deciding which animals to treat and when to administer treatment if it is required.^6^

The faecal egg count (FEC) is an accessible, non‐invasive diagnostic test in which the number of GIN eggs present in a faecal sample is counted to provide an indication of the GIN burden of the animal. Routine FEC in grazing animals, either analysed individually or using pooled samples from a group, can be used to evaluate grazing risks and evaluate anthelmintic efficacy using a faecal egg count reduction test (FECRT). Long‐term monitoring is also particularly beneficial, enabling farmers to evaluate the impact of season on anthelmintic efficacy (as a proxy for changes in species composition, which typically occurs seasonally^7^, ^8^, ^9^). Additionally, FECs can help determine the need for treatment, potentially decreasing selection pressure for anthelmintic resistance, targeting treatment to the optimal time so as to maximise production efficiency and reduce the environmental impact of grazing livestock.^6^, ^10^

Despite the simplicity of the test and its versatility, few farmers use FEC routinely, and very few in the long term. Previous surveys have shown that around half of farmers on both lowland and hill systems used FEC to determine the need for treatment^8^, ^11^ and this is likely an over‐estimation due to the bias in surveyed populations, which may already have awareness of the importance of FEC monitoring.

A possible barrier to the uptake of FEC is the difficulty in interpreting results. As most sheep can tolerate a level of parasitism without production losses, farmers need to interpret results for their flock based on the potential clinical impact. In addition, further limitations such as the inability to morphologically differentiate between pathogenic and non‐pathogenic species, make non‐specialist interpretation challenging. Previous work has shown that complexity, or conflicting advice, is the most substantial barrier to the uptake of monitoring FECs.^12^, ^13^, ^14^ Many farmers conduct testing through their veterinary practices or animal health advisors and thus receive additional information with the results to support decision making. However, FEC testing is also available from specific FEC companies, through new diagnostic tools (e.g., FECPAK G2 or MicronKit^15^, ^16^), and is being conducted on‐farm by an increasing, but still small, number of farmers. In these cases, it is possible farmers may lack professional guidance and support in interpreting test results and the subsequent decision‐making process.

Decision support tools (DSTs) aim to help users interpret complex information by translating it into useful information and/or an effective outcome.^17^ In agriculture, software‐based DSTs are increasingly common in fields such as crop management.^18^ Adoption of precision livestock farming is increasing the development of DSTs for livestock production, with over 40 tools currently available for dairy production^19^ and an increasing number of published tools available to aid sheep production.^20^, ^21^ Tools can be applied to solve challenges at all levels of production, from industry‐level assessments for policy makers to enabling farmers to make specific farm‐level decisions.^22^, ^23^

This project aimed to develop a DST to simplify the interpretation of FEC results for farmers, veterinarians and animal health advisors. Evidence‐based decision making and signposted advice should raise awareness of the need to use anthelmintics responsibly, reducing the selection pressure for resistance while maximising production efficiency in grazing sheep.

## MATERIALS AND METHODS

**FIGURE 1:**
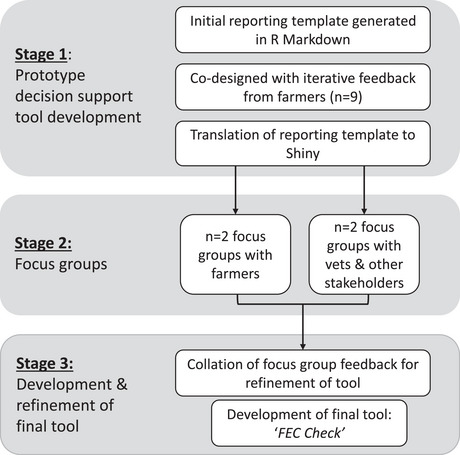
Flow chart of the decision support tool development process.

A DST was developed for the interpretation of FEC results using a participatory‐based approach that engaged with end‐users (livestock farmers, veterinarians and animal health advisors). An overview of the DST development process is shown in Figure 1, and each stage is described further below.

### Stage 1: Conceptualisation

The initial concept of the DST was to create a simple graphical representation of sheep FEC results, using a ‘traffic light’‐style gradient indicating the potential clinical impact of the egg count value to improve understanding and interpretation of diagnostic results for end‐users. The values used to set up the gradient colour change were based on published guidelines for strongyle‐type roundworms of sheep on farms without *Haemonchus contortus* (due to high level of egg shedding of this species^24^), as outlined by the industry group ‘Sustainable Control of Parasites in Sheep’ (www.scops.org.uk/advisers‐technical‐info/).^2^ This also excludes *Nematodirus* spp., which are reported separately due to its different life cycle and clinical significance.

Initially, this concept was developed as a PDF reporting template designed using RMarkdown^25^ to report monitoring FEC results. This was then extended to calculate anthelmintic efficacy (with a 95% confidence interval), where FECRTs were conducted. This aimed to aid decision making and enable simple assessment of anthelmintic efficacy. An example of this reporting template can be found in Supporting Information S1.

Nine farmers conducting FEC testing as part of a larger project were consulted throughout stage one of the development process to provide iterative feedback on the usability of the report and guidance on the design of the visualisation to ensure that it was fit for purpose. This template was used consistently for 12 months prior to further development of the prototype DST. Ethical approval for the nine farmers involved in the conceptualisation was obtained through the Moredun Research Institute's Animal Welfare and Ethical Review Body (ref. NARF03/23).

Following this initial period, a web‐based DST was developed to offer a version of the R Markdown template with an accessible graphical user interface (GUI) designed for wider use by end‐users of FEC results, such as farmers, veterinarians and livestock health advisors. The prototype tool was developed using Shiny,^26^ a web application framework, which negated the need for specialist knowledge to produce reports. The design of the tool considered the desirable attributes on the checklist for good design of DSTs in agriculture,^17^ which contains 15 points, including performance, ease of use, cost and relevance to user.

**FIGURE 2:**
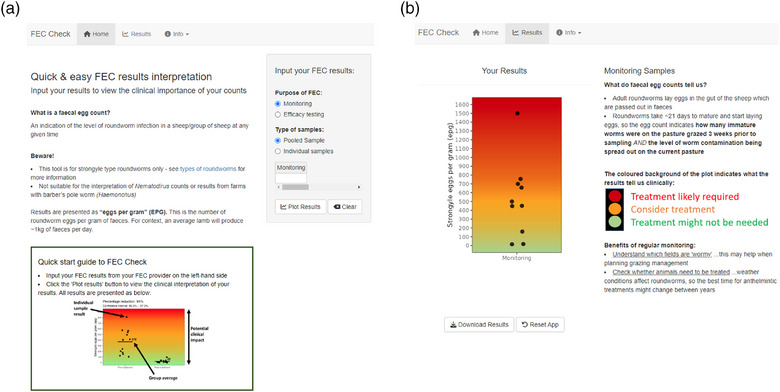
Screenshots from the prototype decision support tool. (a) Graphical user interface for initial data input. Users first select the purpose of the faecal egg count (FEC) testing, then the type and number of samples. The data input table responds to the inputs to display the appropriate data input fields. (b) Visual representation of FEC results. Gradient represents the potential clinical impact of the FEC results based on industry guidelines, with each black dot representing an individual sample result.

The GUI of the prototype DST included options to select the purpose of testing (monitoring or anthelmintic efficacy testing) and type of samples (individual or pooled; Figure 2A), which subsequently displayed the appropriate data input fields for the creation of the visualisation. This enabled users to directly input data, as opposed to requiring the upload of a data file, which would likely be inaccessible for many users due to time and required technological literacy. The output figure could then be downloaded as an image file by end‐users. The colours used for the traffic light gradient used a lighter shade of green and darker shade of red to increase accessibility for coloblind users and enable greyscale printing of the output figures.

Alongside the output figure (Figure 2B), further information was provided to support the interpretation of the results. For monitoring FECs, information included a traffic light descriptor to show how the colours correlate with the potential need for treatment. Additional information describing how the FEC result relates back to in‐animal infection level and on‐field contamination (‘What do FECs tell us?’) and how FEC testing can be used longitudinally to target anthelmintic usage and plan grazing management (‘benefit of regular monitoring’) were also included. To support the interpretation of efficacy testing results, a table was included explaining how to interpret the percentage reduction (e.g., a reduction of >95% showed that this treatment worked well), and a checklist of factors, which influences the validity of FECRT results, for example, FECs conducted on fresh faecal samples with an adequate pre‐treatment count.

To further support sustainable roundworm control, additional resources with relevant information pertaining to FEC testing and anthelmintic use were collated and simple information pages were developed. Topics included how to collect useful and representative faecal samples, uses for FEC testing, how to pool faecal samples, information on anthelmintic groups, and the common species of roundworms present in the UK.

### Stage 2: Focus groups

Four 2‐hour focus groups were held in January and February 2023 in two geographically distinct locations in Scotland (Midlothian and Perthshire) to examine the need for a DST to improve FEC result interpretation and gather feedback on the DST from a wider audience. At each location, one focus group was held for farmers, and a second for veterinarians and advisors. Splitting the focus groups by stakeholder type allowed for assessment of whether opinion and/or requirements differed between groups, which would inform potential tailoring of the DST to specific stakeholder groups. Focus groups were facilitated by two veterinary parasitologists (L.A.M. and E.G.) that were both involved in the prototype DST development to allow for adequate technical support to be provided to participants if required. A further veterinary parasitologist (J.M.D.) was also present as a notetaker to document group discussions.

Animal health advisors and veterinarians practicing local to the focus group venues were invited by email and encouraged to recruit farmer clients to the sessions for farmers. Additional participants were recruited through the local Moredun regional advisors (a group of industry representatives with local farming networks) and a SAC Consulting advisor. The participants were asked to sign an informed consent form at the start of the focus group meeting, agreeing to the collection of their views and opinions for future development of the DST and publication. The participants also received information prior to the meeting regarding the collection and storage of data and how they could opt out of the study.

At the beginning of each of the farmer focus groups, the participants were asked a set of four structured questions, shown in Table 1, relating to their previous experience with FECs. The data were collected anonymously using keypad clickers and the results were displayed, anonymously, live on a large screen as a foundation for further discussion. For the veterinarians and advisor focus groups, the initial questions were unstructured to facilitate discussion around client use and experiences with FEC. Structured questions were only used in the farmer group, as the authors were interested in quantitative data on FEC use due to the lack of existing data for the purposes of targeting DST development in these areas and for future research purposes. The full discussion guide for both focus groups is available in Supporting Information S2.

**TABLE 1:** Structured questions that were asked to farmers at the focus groups related to previous faecal egg count experience.

Question	Possible answers
Do you currently use faecal egg counts?	Yes No
What do you use faecal egg counts for?	Checking treatments were effective Monitoring infection throughout the season Spot checks to see if animals need treatment
What type of faecal egg count testing do you use?	Pooled Individual animals
Who normally performs the faecal egg count?	Local veterinarian Online or postal provider Agricultural merchant On farm Other

After the initial questions, all the participants were introduced to and given an overview of the features of the prototype tool, they were then provided with a piece of technology, either a laptop, tablet or mobile phone, to access the prototype tool individually. The introduction provided was the same for both the farmer and advisor groups. The purpose of using multiple types of technology was to test useability on different screen formats to emulate different use cases. To test the tool, the participants were provided with mock FEC results of monitoring and efficacy tests and asked to input these results and create a visualisation. The participants were then asked to test the additional features such as the download function and additional resources. Materials were provided for note taking. Facilitators circulated to provide technical support during this time if required.

After approximately 15‒20 minutes to allow all participants to fully test the functionalities (timings varied between sessions due to familiarity with technology), the facilitators chaired a group discussion about user's experiences with the prototype tool. Discussion at all focus groups was largely unprompted. However, additional prompts were used by facilitators if discussion became too tangential or to cover topics that were not naturally raised. The list of prompts followed by each discussion is shown in Box 1. Written notes of discussion points, including verbatim accounts, were taken by the notetaker. The participants were also given the opportunity to provide comments in a written format if preferred.

**Box 1: Prompts for discussion when evaluating prototype decision support tool**

- What do you like about the tool so far and why?
- What do you dislike about the tool so far and why?
- Who do you think would use it?
- How do you think the tool would be used in practice?
- Is there any additional information that you think needs to be included with this tool?

The results from the four focus groups were coded using an inductive approach in NVivo 12 by an experienced social scientist (K.L.) independent from the focus group facilitators. The codes are available in Supporting Information S3.

### Stage 3: Development and refinement of final tool

Refinement of the prototype DST was based on feedback attained from the focus groups. The participants suggested changes and additional functions, which were listed, then ranked by priority (a qualitative assessment of frequency of mention and emphasis given to features at the focus groups). Functions were further ranked by ease of incorporation into the tool. Iterative development of the DST was performed to incorporate as many participant suggestions as possible.

The final DST was built by Basestation (Edinburgh, UK) and hosted through the Moredun Foundation website.

### Uptake of DST

The final DST, ‘FEC Check’, was launched on 1 June 2023, and disseminated to industry stakeholders through social media, agricultural events, articles in local and national agricultural presses, industry stakeholder groups, veterinary students and Moredun Foundation members. The DST was also disseminated to the scientific community at academic conferences and reports generated for funding bodies.

To evaluate the uptake and use of FEC Check, analytics data from the first 12 months (1 June 2023 to 1 June 2024) were collected using Google Analytics. Specifically, data on the number of users per week and the number of visits per page were collected and visualised in R v4.3.1 and RStudio 2023.06.2+561 ‘Mountain Hydrangea’^27^ using the packages ‘ggplot2’ v3.4.0^28^ and ‘cowplot’ v1.1.1.^29^

## RESULTS

In total, 33 participants attended the focus group sessions, comprising 17 farmers, two large animal veterinarians and 14 livestock health advisors.

### Current use of FEC by focus group attendees

All participants who attended the focus groups were familiar with FEC testing and 77% of 15 farmers (with two non‐responses) used FEC testing on their farm (Table 2). Most farmers used FEC for spot checks (88%) to determine whether anthelmintic treatment was necessary at a singular point in time, but around half of the respondents also used FEC for monitoring over time (53%) or to test anthelmintic efficacy (41%).

**TABLE 2:** Previous experience and use of faecal egg count (FEC) testing, as self‐reported by farmers (*n* = 17).

	Responses	
	Proportion of total participants (%)*	Number of participants (*n*)
FEC use		
Yes	77%	13
No	12%	2
Reason for use		
Monitoring	53%	9
Efficacy	41%	7
Spot check	88%	15
Samples used		
Pooled	94%	16
Individual	41%	7
FEC provider		
Veterinary practice	71%	12
Online	12%	2
Agricultural merchant	6%	1
At home	47%	8
Other	6%	1

* Not all participants responded to all the questions. Multiple selections could be made for all questions except FEC use.

While participants were not specifically asked about additional uses or benefits of FEC testing, the farmers mentioned using FEC for selecting replacement breeding stock, understanding challenges facing their stock and as part of good pasture management. The veterinarians and advisors also agreed that FEC was a useful tool and that they were being increasingly used to investigate suspected anthelmintic resistance.

### Current reporting of FEC results

The participants from the farmer focus groups had experience of receiving FEC results from a variety of sources. When asked about how FEC results were communicated to them, the farmers described different levels of engagement and feedback. In the first focus group, most participants agreed that their veterinarians were engaged, stating, ‘[I] get numbers back, information on what I should do, for example, what drench and animal dosing history’, and for some farmers, this was ‘usually followed up with the full report being emailed’. However, this was not a unanimous experience. Some farmers felt that they were provided limited information back, stating ‘the vet only tells me whether or not I should drench, I am not given any numbers back’, and that they needed to ‘dig for information’ to interpret the results further.

Veterinary engagement was discussed as a challenge for receiving more detailed feedback with their results. One farmer stated that ‘vets who are engaged prefer to have farmers who are also interested and engaged’.

At the veterinarian and advisor focus groups, the veterinarians stated that they would conventionally phone clients or send them a text message with their results, but there was a consensus among both groups that full results reports, if provided, were typically not utilised by farmers.

### Barriers to FEC uptake

#### Timeliness of results feedback

The length of time between faecal sample collection and receiving results was acknowledged as an important barrier to FEC uptake in all focus groups. This was related to both the turnaround time from the FEC being conducted (normally through an external laboratory) and receiving the results. While they believed FEC was valuable, farmers stated they ‘need to know on the day what's wrong and how to react … not wait for FEC results from [their] vet for advice’.

The veterinarians added that in practice it was not always feasible to conduct FEC in‐house due to lack of time, with most of the demand for FEC testing occurring during busy periods, stating that they ‘could only do FECs on quiet days and it is not the most effective source of income’. In addition, they stated that it would require staff to develop additional technical skills and knowledge, which can easily be outsourced to external laboratories.

Subsequently, there was discussion between farmers about conducting FEC on‐farm, adopting a ‘do it yourself’ (DIY) approach with farmers stating, ‘those that do FEC at home do it because they don't want to wait 7‒10 days for results’. Farmers who undertook FEC testing at home (*n* = 8; Table 2) agreed that they found the process easy and worth the set‐up costs and ‘gets you thinking’ about the results more. Farmers who did not conduct FEC testing on‐farm were concerned about how time consuming it would be to perform.

#### Lack of perceived need

The veterinarians felt that FEC uptake may be limited due to established habits within the farming industry. For example, farmers practicing ‘calendar farming’, treating animals on set dates rather than evidence‐based approaches, or treating individual animals based on clinical indicators such as low weight gain. The veterinarians and advisors attributed this to farmers underestimating the prevalence of anthelmintic resistance, and that anthelmintics are ‘too cheap’; thus, farmers may not be inclined to wait for FEC results before treating their animals. There was no mention of a lack of perceived need by either of the farmer groups.

#### Knowledge gaps

Among the farmer groups, there was some confusion regarding aspects of FEC testing such as appropriate post‐treatment sampling intervals for assessing anthelmintic efficacy. One participant stated that they had ‘lost confidence in FEC’ after observing clinical signs of parasitic gastroenteritis shortly after receiving a low FEC result from the same group of animals. Further discussion identified a lack of appreciation for the impact of substantial changes in climatic conditions on infection risk (with a large amount of rainfall after a very dry period leading to a period of mass egg hatching). The veterinarians and advisors felt there was also a general lack of communication between themselves and farmers regarding the benefits of FEC which may be limiting uptake.

### Prototype DST feedback

Upon introduction to the prototype DST, all the participants liked the visual aspect of the tool. The farmers felt that the DST could support the relationship between them and their veterinarians, stating that it would be ‘helpful to talk over with vets for treatment plans’. Equally, the veterinarians added that it was a ‘great visual understanding for farmers’ and would be a ‘good tool for vets and advisors to track how many FECs are being done’. The farmers also felt strongly that this type of tool could help to develop a deeper understanding of what is happening on farm, stating that ‘we as farmers need the ability to turn data, into information, into knowledge and that is what an app should be able to do’. However, it was acknowledged that the benefit farmers would gain from the DST was dependent on it being used effectively.

It was also reiterated throughout all focus groups that simplicity and ease of use would be critical to uptake and that requiring farmers to input non‐essential metadata along with their results may actively discourage uptake.

Downloading results was seen as an important factor for users to be able to build a historical picture that could be used to inform future decision making, with farmers stating, ‘if I have the app I am more likely to do FEC more often if I can build a bigger history which will provide me with a better understanding’.

Furthermore, several participants felt that the DST would be particularly beneficial for farmers conducting FEC on‐farm and provide additional resources to understand the implication of their results where access to engaged veterinarians or advisors was not possible.

“;For farmers doing their own FEC it will be fab and would encourage those not doing [it] to do their own as [they get] quicker results.”

“;[I] think it is great and could tie in with home testing. [It] can help to select which animals are good tups, need culled, etc”

Due to the good agreement in the feedback received from the farmer and veterinarian/advisor focus groups presented above in terms of how the tool would be used by each group (e.g., retention of FEC data for health planning), it was established there was not a need for different tools for these two groups.

The participants were able to use the DST successfully on each of the different devices, although those using smaller screens (e.g., smartphones) noted that buttons and text boxes were small, making data input more challenging. Table 3 summarises suggested amendments and additional functions that focus group participants believed would improve the DST and promote uptake by stakeholders.

**TABLE 3:** Features proposed by focus group participants, for incorporation into the decision support tool (DST), ordered by priority assigned based on focus group feedback.

Feature	Priority	Ease of incorporation
Improve accessibility (e.g., button and text input box sizes)	High	1
Option for post‐treatment drench check	High	1
Option for pooled testing	High	1
Inclusion of *Nematodirus* spp. visualisations	High	1
Improved smartphone compatibility	High	1
Alerts for post‐treatment sample collection	High	3
Tutorial video of at home FEC testing	Moderate	1
Additional information boxes (e.g., group name) for downloading	Moderate	1
Further ‘additional information’ pages	Moderate	1
FEC and anthelmintic efficacy data collection for national‐level passive surveillance	Moderate	2
Offline capability	Moderate	3
Extend to other host species (e.g., cattle)	Moderate	2
Longitudinal data option	Moderate	2
In‐app data storage/retention for historic comparisons	Moderate	2
Log‐in with farmer/veterinarian interface	Low	3
Integration to existing flock management tools	Low	3
Benchmarking results against other users	Low	3
Option to add electronic identification or average daily live weight gains	Low	2

*Note*: Ease of incorporation—scale from 1 to 3, where 1 represents features that would require minimal additional resources to incorporate immediately or at least within the subsequent months and 3 represents features that would require significant and/or fundamental changes to the DST thus would require significant resources to implement.

Abbreviation: FEC, faecal egg count.

### Final decision support tool and initial uptake

The final DST, *FEC Check*, was launched on 1 June 2023, and it is freely accessible to end‐users (https://app.moredun.org.uk/fec/sheep). The tool is compatible with multiple operating systems and device types, including smartphones, prioritising ease of use and accessibility.

**FIGURE 3:**
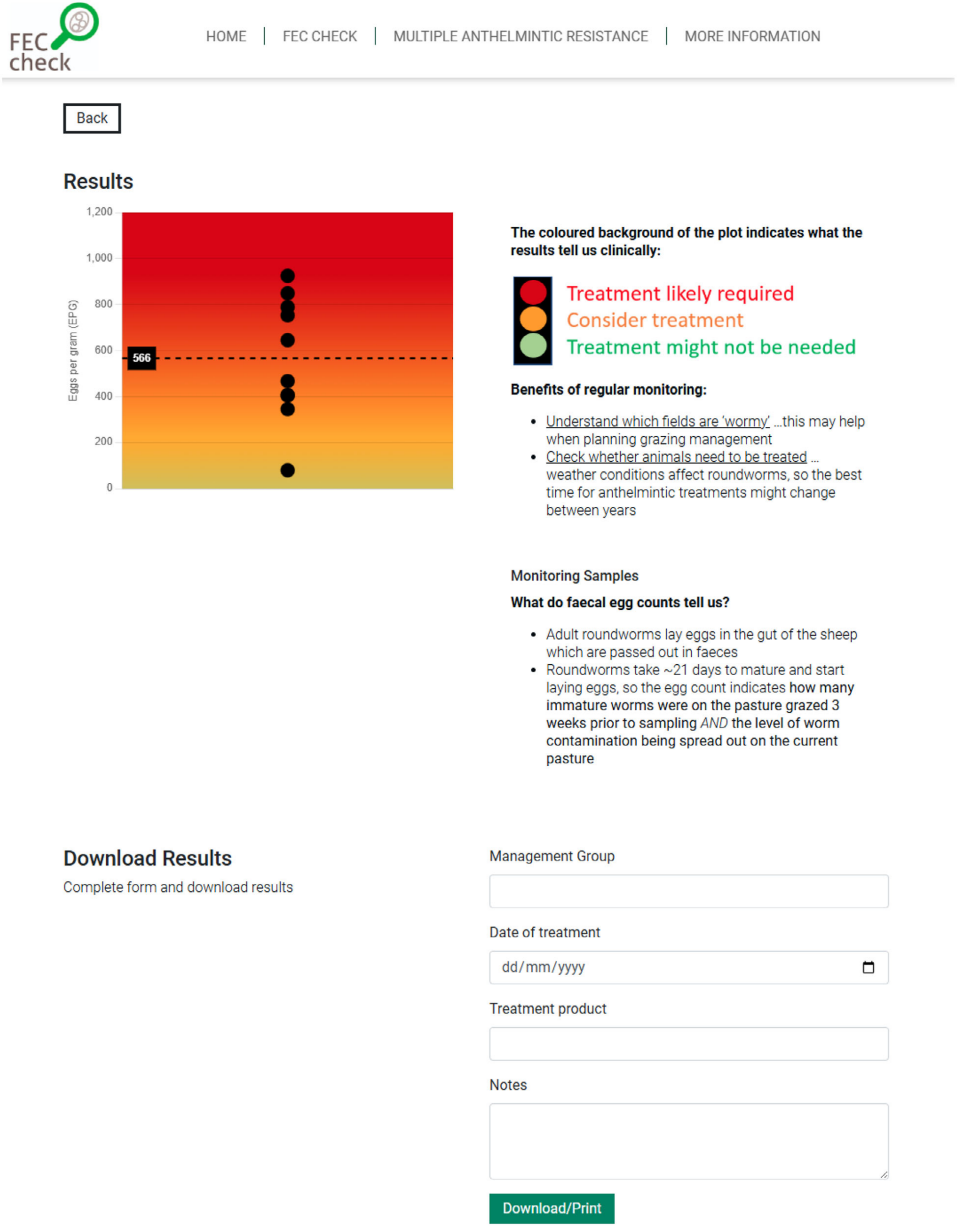
Final decision support tool output visualisation using the example of a 10 individual monitoring samples for roundworms with a mean of 566 EPG. This version incorporated some of the changes requested at the focus group sessions. Improvements demonstrated here include improved accessibility and additional information boxes for downloads.

The ability to visualise *Nematodirus* spp. counts was incorporated by asking users to specify the roundworm species they were testing for on the DST's data input page, and a ‘post‐treatment’ drench check (where the effectiveness of a treatment is checked by conducting post‐treatment FECs without having performed pre‐treatment FECs to enable the calculation of anthelmintic efficacy) was added as an additional option for purpose of testing. Relevant information relating to each of these new pages was also added. Furthermore, the ability to download results was updated to allow users to optionally specify sample information, including the management group, date of treatment, treatment product and add further notes, as shown in Figure 3. If provided, this information was included on the printout or download of the DST output.

**FIGURE 4:**
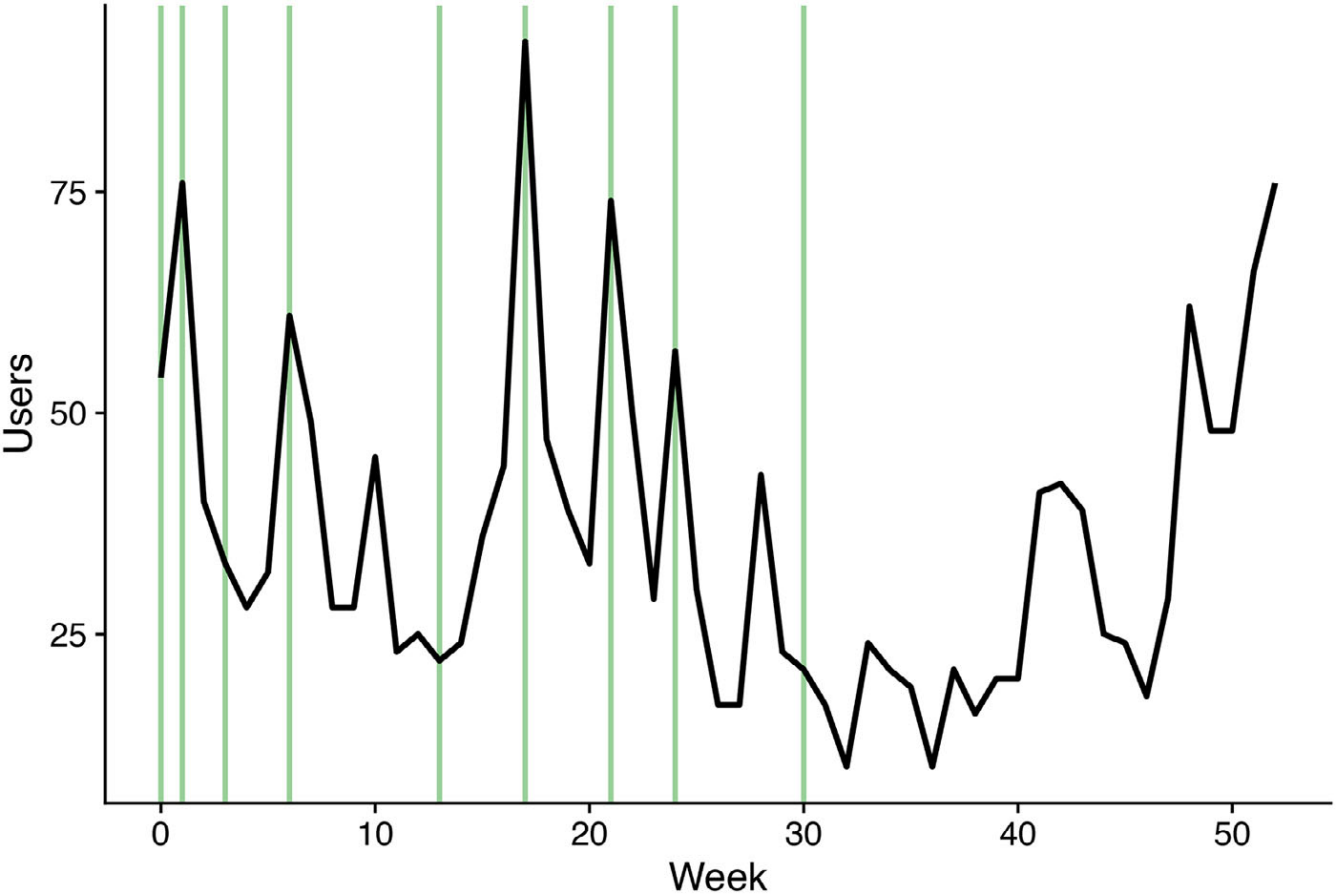
Number of users accessing ‘FEC Check’ decision support tool per week across the first 12 months after its launch on 1 June 2023. Green vertical lines represent dissemination events such as agricultural shows, academic conferences and articles in the farming press.

In the 12 months since its launch, the FEC Check DST was visited 1916 times, averaging 36 users per week (range: 10‒92 users). Figure 4 demonstrates the number of visitors per week from the launch. Initially, spikes in engagement largely coincided with dissemination events undertaken by the authors (illustrated by the green vertical lines). Over winter, from approximately weeks 25‒41, engagement was generally under 25 users per week. However, there was substantial upward trend in the number of users from week 45 onwards (11 April 2024) without the occurrence of any dissemination events.

Excluding the homepage and more information landing page, FEC Check received 3920 page views (mean = 2.05 pages per visit). Of these, over three‐quarters of views occurred on the FEC Check DST (for users to input FEC results to produce a visual representation of the counts; Table 4), representing an average of 2.05 views per user. Additional information pages received 15.4% of the total views, with the DIY FEC page accounting for over half of these views (Table 4).

**TABLE 4:** Number of views received on faecal egg count (FEC) Check decision support tool (DST) website in the first 12 months post‐launch by page, excluding landing pages, from 1 June 2023.

Page	Views	Proportion of total views (%)
FEC Check DST	3118	79.5%
Multiple anthelmintic resistance decision tree	198	5.1%
Additional information pages	604	15.4%
DIY FEC	332	8.5%
Types of roundworms	77	2.0%
Uses for FEC testing	56	1.4%
Collecting good quality faecal samples	43	1.1%
Understanding anthelmintics	39	1.0%
Anthelmintic resistance	23	0.6%
Pooled testing	34	0.9%

Abbreviation: DIY, do it yourself.

## DISCUSSION

FECs are an accessible tool for assessing GIN infections in grazing livestock to target treatments and monitor anthelmintic efficacy. However, it is important that farmers and other stakeholders interpret results effectively and accurately to prevent unnecessary anthelmintic use, which can increase selection pressure for anthelmintic resistance. This study used a participatory approach to develop a DST, FEC Check, for the interpretation of FEC results in sheep using a simple visual of potential clinical impact. Paired with relevant information and resources, this tool could standardise results reporting across the industry, allowing farmers to make decisions for their flock based on best‐practice guidelines.

The development of the DST was motivated by informal remarks from farmers at knowledge exchange events and participation in other studies of varying levels of feedback being provided with FEC results. These reports were supported by the results from the focus groups, with some farmers stating that they only received a treatment decision without any numbers or vice versa while others received a more detailed breakdown of the results and how this was fed into the suggested treatment recommendation. The mixed experiences of producers may be in part due to limited resources within veterinary practices (with FEC testing often aligning with busy periods) or due to a lack of engagement. FEC Check provides the required additional support for farmers, particularly for those conducting on‐farm FECs.

The FEC Check visual was designed to indicate the magnitude of the FEC while avoiding defined threshold values for triggering anthelmintic treatment. There are several factors, including presence of other diseases, nutrition and immunity, which can modify the impact of parasitism on an animal's productivity, health and welfare.^30^ The design encourages users to consider recent management decisions for the interpretation of borderline values and urges users to engage with veterinarians and/or animal health advisors to discuss their individual situation. The advice administered by the DST is broadly useful but variation between farms, such as climate or farm management (which are not incorporated into the current DST), could influence farm‐specific advice. Therefore, the aim of the DST is to empower farmers to engage in discussions with veterinarians and advisors about sustainable parasite control by interpreting FEC results and providing accessible information with which they can frame specific questions. Long‐term use could also be particularly beneficial for flock health planning.

Research has shown that veterinarians are a trusted information source for more than 80% of farmers,^31^ highlighting the importance of veterinarian‒farmer interactions and the influence these relationships have on on‐farm decision making. Veterinary engagement could have a significant impact on the success of the tool, and wider uptake of sustainable parasite control on farm. FEC Check was not designed to replace veterinarian‒farmer interactions but rather to support discussion by aiding the understanding and usability of diagnostic test results and providing the necessary additional information around the topic. With this knowledge, farmers can feel informed and better equipped to discuss the results with animal health professionals and are more likely to make evidence‐based treatment and management decisions. It was encouraging that focus group participants agreed that FEC Check could be positive for the farmer/veterinarian relationship, but unfortunately, few veterinarians took part in the focus groups (two veterinarians attended). This lack of engagement could be due to the meetings being conducted during the working week and the lack of available reimbursement for their time due to the restricted research budgets, or it could reflect opinions on the perceived need for this type of DST or the value of FEC. Given the anticipated influence of vet opinion on farmer decision making, it may be helpful to undertake further work to understand the opinions of veterinarians on the DST and FEC more widely.

Previous research suggests that less than half of sheep farmers currently use FEC monitoring.^11^ The use of FEC by focus group participants here was higher (77%), although this may be biased resulting from self‐selection for the meeting. The veterinarians and advisors did, however, report a perceived ‘lack of need’ for FEC testing within the wider industry. With previous work showing the use of FEC has been triggered by the presence of anthelmintic resistance on farm^12^ and a high proportion of farms estimated to have anthelmintic resistance,^3^, ^8^, ^32^ perceived lack of need may be due a lack of knowledge regarding anthelmintic efficacy status. The launch of agricultural grants for farmers to use evidence‐based approaches to improve farm animal health and welfare,^33^ which includes FEC testing, may help to promote uptake of FECs. In particular, this may engage farmers who have not previously used FECs. The development of FEC Check for the interpretation of FEC results may therefore be a particularly timely resource for the industry.

Additional knowledge gaps were identified on the appropriate timing of FEC for efficacy testing and the impact of weather patterns on the GIN lifecycle. A farmer involved in the focus group reported that they ‘lost confidence in FEC’ because of unexpected results following a period of high rainfall after a prolonged dry spell, resulting in a mass hatch event and higher FEC results. Promoting the use of testing is important but ensuring producers understand the benefit and recognise when to seek further advice is key to promoting routine use. This finding further emphasises the importance of accessible background information and guidance on how and when to implement testing and when to seek further advice to ensure that results are useful and support evidence‐based decision making.

Timeliness of results reporting from FEC providers was identified at focus groups as a major barrier to the adoption of testing. Over 80% of participants in the farmer focus groups used an external service for FEC testing (e.g., veterinary practice or agricultural merchant), which participants reported could take up to 10 days to receive results. Farmers who cannot easily gather animals to sample, or have limited opportunities to handle animals, may then be more likely to treat than wait for results. Consequently, it was perhaps unsurprising that several farmers were interested in undertaking FEC testing on‐farm and the ‘DIY FEC’ information page received over half of the views of the FEC Check information pages within the first 12 months. The inclusion of additional information on the method for collecting good samples will be useful in this regard, improving the reliability of the results from DIY FEC testing.

Throughout the focus groups, simplicity was identified as a key factor that would drive uptake of the DST. Most of the features implemented in the final DST were suggestions deemed as high priority by the focus group participants (Table 3), which would offer substantial value to the DST without compromising the overall simplicity. As a result, features in the final DST focused on improved accessibility with larger buttons on smartphones and tablets, a smartphone‐friendly format and additional data input options (e.g., post‐treatment drench checks and pooled testing). The DST could be expanded in the future to process FEC results from other livestock species, but the overall simplicity of design should be prioritised to safeguard its use by new users of FEC testing and users with lower technological literacy.^34^

While simplicity was the main priority, there were more complex feature suggestions made, such as in‐app data storage, longitudinal data visualisation, log in with a farmer/veterinarian interface, or incorporation into larger flock‐management software packages. These suggestions have the potential to provide a greater depth of value from routine FEC monitoring and efficacy testing. However, they would require substantial changes to the framework of the existing tool and would affect the simplicity of the current version. It is possible these features could be included in an upgraded version of the DST while maintaining the base version presented here. While the intention was to maintain the base version for free, it is likely that an upgraded version would need to have an associated fee (either one‐off or subscription‐based) to cover the running costs of data storage. This could, however, also enable continued development of the tool that would not otherwise be possible without seeking further funding.

The ability to retain FEC results for future reference was viewed as a key feature, although the prototype DST does not store data, users can download results. In each group, the participants expressed that they would like to be able to store data within the DST itself to create longitudinal reports and summaries for use in future grazing seasons. While this was not possible in the final DST presented here, the download function was improved to allow farmers to add metadata such as management group and treatment date. This will allow farmers to build a database of results by downloading or printing reports, without the need to store data. This could be particularly useful for sharing results with their veterinarian. With further resources, in the future, it may be possible to allow farmers to log in to an interface and store results to create longitudinal reports of FEC results and anthelmintic resistance. It would also be of interest in the future to explore how the use of the FEC Check DST has influenced the uptake of diagnostic testing and/or decision making around anthelmintic treatment and efficacy testing on farm.

The DST was designed to visualise roundworm FECs for farms where *H. contortus* was not present, as is typical on UK sheep farms. However, recent surveillance has demonstrated that clinical disease caused by *H. contortus* is increasing in the UK.^35^ Consequently, the DST may need to incorporate gradients for the interpretation of FECs where *H. contortus* is present to maintain relevance to users. Alternatively, it may be more appropriate to signpost to resources on FAMACHA testing, an anaemia scoring index.^36^ While potentially future proofing the DST for UK users, the inclusion of an option for farms where *H. contortus* is present would also provide greater international relevance to the DST, which would increase its potential adoption. However, if this is implemented, care will have to be taken to ensure that any additional resources are internationally relevant.

The number of available DSTs for the livestock sector has grown significantly in recent years, with almost 400 tools aimed at farmers and livestock health advisors.^17^ All advisors and 49% of farmers used DSTs regularly, but the availability and uptake of DSTs were not equal across the different farming sectors.^17^ The disparity in the level of monitoring already in place in different farming types has likely driven this variation (e.g., high level of monitoring and production statistics in dairy compared to many sheep enterprises). The wide array of DSTs all aim to utilise on‐farm data, which are already being generated to optimise farm management and healthcare interventions. FEC Check has been developed to help support data utilisation for grazing livestock, a sector currently under‐represented by available DSTs. It is difficult to compare uptake of FEC Check with other DSTs currently available, as the targets of each are different and usage data of other tools are not readily available. For FEC check, uptake over the first 6 months was largely driven by dissemination events. These events included agricultural shows, mailings to more than 10,000 Moredun Foundation members, and presentations to audiences, which included farmers, veterinarians and academics. After this initial period of dissemination, the number of users in spring of 2024, when farmers may consider administering their first anthelmintic treatments to lambs, increased organically. This may suggest that the use of the DST, either the tool or its resources, may be being used as part of routine parasite management on some farms. This is a promising finding, both for the adoption of the tool, and in promotion of evidence‐based parasite management. Continued growth of the DST will likely be driven by further dissemination events but may also benefit from peer‐to‐peer recommendation.^37^

## CONCLUSION

This study describes the development and early adoption of the novel DST FEC Check, a timely resource to standardise results reporting of FECs for farmers and the wider industry. This tool was co‐developed with input from multiple stakeholder groups to ensure that it aligned with industry needs and was presented in an accessible, fit‐for purpose manner. The uptake observed within the first year of this tool being launched demonstrates the benefit to the iterative, co‐development approach taken. Ultimately, the use of FEC Check could help promote the uptake of FEC testing by producers as a part of a stepwise approach towards the adoption of sustainable parasite management in the sheep industry.

## AUTHOR CONTRIBUTIONS

*Conceptualisation, formal analysis, funding acquisition, investigation, writing—original draft and writing—review and editing*: Eilidh Geddes. *Funding acquisition, investigation and writing—review and editing*: Andrew Duncan. *Formal analysis, funding acquisition and writing—review and editing*: Kate Lamont. *Investigation and writing—review and editing*: Jade M. Duncan. *Funding acquisition and writing—review and editing*: Dave J. Bartley. *Conceptualisation and writing—review and editing*: Neil Sargison. *Conceptualisation, funding acquisition, investigation and writing—review and editing*: Fiona Kenyon. *Conceptualisation, funding acquisition, investigation, writing—original draft and writing—review and editing*: Lynsey A. Melville.

## CONFLICT OF INTEREST STATEMENT

The authors declare they have no conflicts of interest.

## ETHICS STATEMENT

Ethical approval for the focus groups was obtained through the Moredun Research Institute's Animal Welfare and Ethical Review Body (ref. NARF04/22).

## Supporting information



Supporting Information

Supporting Information

Supporting Information

## Data Availability

The data that support the findings of this study are available from the corresponding author upon request.
