# Supplementary material for: FEC Check: Development of a decision support tool to aid interpretation of gastrointestinal nematode faecal egg counts in sheep
Source: Vet Rec. 2026 Jan 6;198(9):e373–84. doi: 10.1002/vetr.70221 (PMC13133759; doi:10.1002/vetr.70221)
Supplement: Supplementary file 1 — Supporting Information [file VETR-198--s002.pdf]

Supplementary Material 1: Example of a mock report generated using the initial reporting template

FEC Results Report

Lambs

Report generated: 16 January, 2023

Treatment Date: 2022-12-23 **Treatment product:** Zolvix

Sampling interval: 14 days **Active compound:** Monepantel

Interpreting this report

Faecal egg counts (FECs) are a monitoring tool that provide a snapshot of the parasite challenge faced by the sampled group at the time of sampling. The number of eggs present is a useful guide for timing anthelmintic treatments, testing the efficacy of treatments, and estimating pasture contamination. FEC results should be considered together with the grazing and treatment history, clinical signs and performance. These factors should be discussed with your vet or adviser before making a treatment decision.

The results here are presented as as the number of eggs per gram (epg) of faecal material, with each point representing the result of an individual animal. The horizontal bar and number is the average number of eggs per gram of faecal material for that group. The gradient background of the strongyle-type eggs figure is shaded with a traffic light style gradient to aid the clinical interpretation of the results in line with the current SCOPS guidelines on farms where *Haemonchus contortus* is **not** present. There is no gradient shading for the *Nematodirus spp.* results as there are currently no published guidelines to aid the interpretation of these counts.

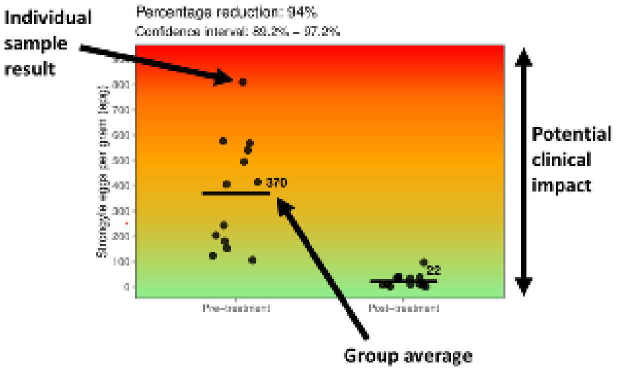

Interpreting the percentage reduction

The percentage reduction is an indication of treatment efficacy which indicates whether the anthelmintic product used was effective.The percentage reduction is calculated based on the reduction of the faecal egg counts post-treatment using *unpaired* samples (different animals samples pre and post-treatment), and with a minimum pre-treatment FEC of 200epg. This calculation also assumes that the correct dosage has been administered to each animal based on their weight using calibrated equipment to administer a product which is in-date and has been stored correctly, and that post-treatment samples have been taken at the correct interval. Providing all of these assumptions are met, the percentage reduction provides an indication of treatment efficacy. The confidence interval is used to validate the percentage reduction due to the differences between the individual samples. The smaller the difference between these two numbers, the more 'confident' we can be that this is the true percentage reduction.

Currently, an anthelmintic treatment is deemed to have been effective when the percentage reduction is 95% or greater. If the reduction is less than this, it suggests the treatment has not worked fully and the reason for treatment failure should be investigated further.

It is important to note that anthelmintic resistance is dynamic and as such the efficacy of treatments will change dependent on the parasite species present at the time of treatment. Particularly where a partial reduction is observed, resistance may be only to single species. Therefore a treatment which was not fully effective early in the season, may be more effective later in the year.

Strongyle results

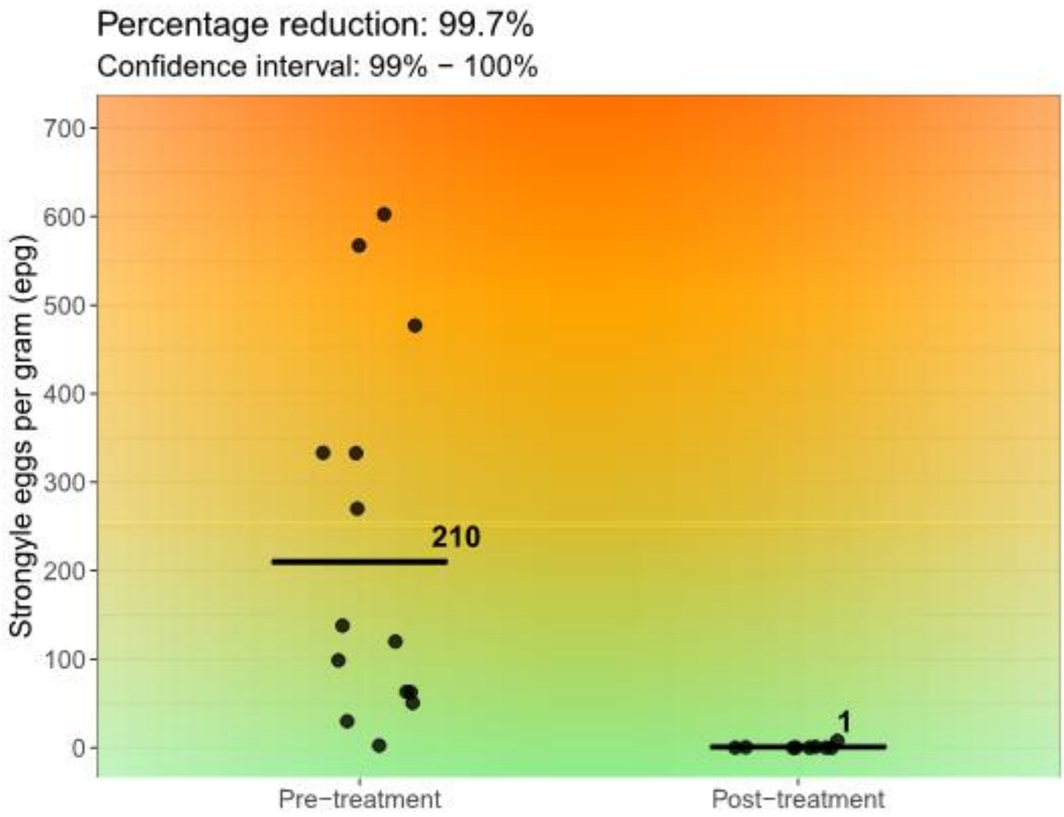

Sample	Pre-treatment (eggs per gram)	Post-treatment (eggs per gram)
1	270	0
2	0	0
3	99	0
4	30	1
5	3	0
6	51	0
7	63	0
8	603	0
9	138	0
10	333	0
11	477	8
12	333	0
13	567	0
14	120	1
15	63	0
**Average**	**210**	**1**
