# Supplementary material for: FEC Check: Development of a decision support tool to aid interpretation of gastrointestinal nematode faecal egg counts in sheep
Source: Vet Rec. 2026 Jan 6;198(9):e373–84. doi: 10.1002/vetr.70221 (PMC13133759; doi:10.1002/vetr.70221)
Supplement: Supplementary file 2 — Supporting Information [file VETR-198--s001.pdf]

Supplementary Material 2: Discussion guide for focus group sessions with farmers, vets and advisors. Initials refer to authors.

Discussion guide for FEC Check Focus Group Discussions

Welcome

Consent forms

Brief introduction to the project (LM)

Clicker questions and discussion (LM)

- What is your role?
  - Vet
  - Livestock health advisor
  - SQP/RAMA
  - Farmer
  - Other

- Do you use faecal egg counts currently?
  - Yes
  - No

- What do you use FEC for?
  - Checking a treatment was effective?
  - Monitoring infection throughout the season
  - Spot checks to see if animals need to be wormed

- What type of FEC do you typically use?
  - Pooled
  - Individual animals

- Who normally performs the FEC?
  - At home
  - Local vet
  - Online/postal provider
  - Agric merchant
  - Other

- What information do you get back?

  - Do you seek additional information to help make treatment decisions? Where from?

- Do you feel well-informed?

- MAR: If you discovered that one or more products were not working effectively, would you be confident in knowing what your control options were and where to find additional information?

Introduce the prototype app and how it works (EG)

Breakout to test app

monitoring/efficacy/additional pages

Discussion about the app

- Likes/dislikes

- Is this something you could envision using yourself or would be useful for other farmers?
  - How do you think you/others would use it?
  - Desktop/mobile use?
  - Monitoring or efficacy?
  - Is there anything we could do to promote use?
    - Social media reminders
    - Advertising at agric merchants?

- FRAMEWORK questions – e.g. design/language/visuals/understanding
  - Was the graph easily understandable?
    - How could we improve this? Video explanation?
  - Language: efficacy testing/monitoring/interpretation
  - Visuals
  - Front page with info then click into the app?
  - Content?
  - Download function
    - Ability to input text/format

- Additions and future aspirations
  - Storage and long-term trends
  - Linked with management software
  - Decision support on control of cases of Multiple Anthelmintic Resistance

Dissemination

Where do you currently get information from?

- Vet
- SQP
- Websites
- Social media
- In-person farm events
- Agric shows/events
- Webinar
- Friends/family/other farmers
- Agricultural press
- Other

How do you think we would be best to disseminate this type of app?

Would you be interested in beta-testing the app?
