# Supplementary material for: FEC Check: Development of a decision support tool to aid interpretation of gastrointestinal nematode faecal egg counts in sheep
Source: Vet Rec. 2026 Jan 6;198(9):e373–84. doi: 10.1002/vetr.70221 (PMC13133759; doi:10.1002/vetr.70221)
Supplement: Supplementary file 3 — Supporting Information [file VETR-198--s003.docx]

**Supplementary Material 3:** Codebook generated in NVivo for qualitative data analysis

| Name of node | Files | References |
| --- | --- | --- |
| Ad hoc comments | 2 | 6 |
| APP | 3 | 7 |
| Good aspects of the app | 4 | 19 |
| Negative aspects of the app | 4 | 14 |
| Potential benefits | 4 | 17 |
| Questions | 3 | 11 |
| Suggestions | 4 | 55 |
| Dissemination | 1 | 1 |
| Communication | 4 | 10 |
| How | 4 | 15 |
| Timing | 2 | 2 |
| FECs | 2 | 2 |
| FEC info currently | 3 | 5 |
| Questions | 1 | 1 |
| Receiving FEC results | 3 | 6 |
| Reporting of FEC results | 2 | 4 |
| challenges in reporting results | 3 | 4 |
| Use of FECs | 4 | 16 |
| benefits of knowing | 3 | 6 |
| Negs and Barriers to use of FECs | 4 | 13 |
